# Mezcal: A Review of Chemistry, Processing, and Potential Health Benefits

**DOI:** 10.3390/foods14081408

**Published:** 2025-04-18

**Authors:** Sandra Victoria Ávila-Reyes, Antonio Ruperto Jiménez-Aparicio, Guiomar Melgar-Lalanne, Fernanda Sarahí Fajardo-Espinoza, Humberto Hernández-Sánchez

**Affiliations:** 1 Centro de Desarrollo de Productos Bióticos, Instituto Politécnico Nacional, Yautepec 62731, Mexico; savilar@ipn.mx (S.V.Á.-R.); aaparici@ipn.mx (A.R.J.-A.); 2 Instituto de Ciencias Básicas, Universidad Veracruzana, Xalapa 91190, Mexico; gmelgar@uv.mx; 3 Facultad de Ciencias de la Salud, Universidad Anáhuac, Mexico City 01840, Mexico; 4 Escuela Nacional de Ciencias Biológicas, Instituto Politécnico Nacional, Mexico City 07738, Mexico

**Keywords:** mezcal, agave, fermentation, distillation, functional properties

## Abstract

Mezcal is a Mexican alcoholic beverage elaborated by the distillation of fermented maguey (*Agave* genus) juice. In Mexico, there is an extensive variety of fermented beverages that embody many of the cultural traditions of this country. They are associated with environmental factors, naturally occurring microbiota, and the local availability of raw materials. Fermentation processes for the elaboration of ancestral beverages are an antique technology used by ethnic groups since pre-Hispanic times; however, these beverages are currently being studied with renewed attention as a source of prebiotics, probiotics, synbiotics, and postbiotics. An important sector of these products is Agave beverages, such as pulque, tequila, and mezcal. Despite the increasing demand for the last beverage, there are still relatively few studies about the chemistry, biotechnology, and health benefits of mezcal. The main aspects considered in this document are the definitions used in the mezcal industry, characteristics of wild and cultivated *Agave* species and varieties, mezcal elaboration technology (including juice extraction, fermentation, distillation, and aging), and potential health benefits related to mezcal, including prebiotics and probiotics, and bioactive compounds, such as phenolics and alcohol. These compounds can make mezcal a potentially functional beverage when consumed moderately.

## 1. Introduction

Fermented beverages are ethnically and socially accepted products widely used for consumption, recreation, and religious or ceremonial purposes. The case of alcoholic beverages has a particularly widespread interest due to their nutritional significance and the feeling of pleasure, which, for many people, remains the single most common motivation for drinking these products [1].

Two main tendencies can be detected in the current research on traditional fermented beverages in Mexico. The first one includes biotechnological approaches to identify and promote functional products containing prebiotics, probiotics, postbiotics, and synbiotics. The second trend is related to *Agave* beverages produced only in Mexico, such as pulque and mezcal, which involve biotechnological methods and the traditional administration of different substrates, especially *Agave* species. The rising demand for mezcal appears, so far, to be leading this tendency [2]. In the last 10 years, official production has increased from 0.98 million liters in 2011 to 14.166 million liters in 2022 [3].

### Definitions

The legal definition of mezcal is provided by the Norma Oficial Mexicana NOM-070-SCFI-2016 [4]. This NOM is the standard or law that includes the rules and regulations of mezcal and defines mezcal as the Mexican distilled alcoholic beverage obtained by the spontaneous or controlled fermentation of the juices extracted from the heart (leafless stem) of the maguey or agave plant harvested in the zones of the protected denomination of origin (DOP), including the states of Oaxaca, Tamaulipas, Michoacán, Guanajuato, Guerrero, Durango, Puebla, Estado de México, Morelos, San Luis Potosí, Sinaloa, and Zacatecas, and followed by a distillation process [5,6].

The NOM includes the following specifications:

(1) Raw materials. The raw materials include only the juice extracted from agave plants [wild or cultivated] within the DOP geographical regions. No other fermentation substrates are allowed.
(2) Categories. The NOM includes three categories of mezcal according to the elaboration process.
  (a) Ancient mezcal. The elaboration of ancient mezcal involves cooking the stem in rustic ovens dug in the ground, mashing the cooked stem with mallets or in stone mills driven by mules or donkeys, fermentation in pottery or wood vessels after water addition, and distillation of the fermented must in fire-heated clay containers.
  (b) Artisanal mezcal. The preparation of artisanal mezcal includes baking the stem in ovens excavated in the ground or cookers with masonry walls, pounding the cooked heart with hammers or in stone or trapiche mills, fermentation in pottery or wood vessels after adding water, and distillation of the fermented juice by means of fire-heated clay, copper, or stainless steel vessels.
  (c) Mezcal. The production of mezcal comprises cooking the stem in ovens dug in the ground or with brickwork walls or autoclaves, crushing the baked stem with mallets or in stone, trapiche, or electric mills, fermentation in wood vessels, masonry vats, or stainless steel tanks, and purification of the fermented must using copper or steel stills or continuous distillation equipment.
(3) Classes or styles.
  (a) Joven or white. Joven or white is an unaged mezcal that is ready for sale after the distillation process.
  (b) Aged in glass. Aged in a glass mezcal is stabilized inside glass bottles for over a year in dark cellars.
  (c) Reposado. Reposado means “rested” in Spanish, and this mezcal rests in wood barrels for no less than two months and no more than one year. Mezcal reposado is caramel in color.
  (d) Añejo. Añejo means “aged” in Spanish. Mezcal añejo is aged in 1000 L or less wood barrels for at least one year, which gives it a dark caramel color.
  (e) Abocado con. Abocado con or “flavored with” mezcals are flavored or infused. This can include flavors from maguey “worms,” damiana, lime, orange, mango, and honey. Other fruits, herbs, and caramel are also common additions.
  (f) Destilado con (distilled with). In the case of destilado con mezcals, certain ingredients are added during distillation to add flavors. The most common ingredients include chicken or turkey breast (“pechuga” style), rabbit meat, mole sauce, plums, etc.

All mezcals must have between 35 and 55° GL at 20 °C.

## 2. Agave Characteristics, Cultivation, and Production

Wild and cultivated agave (commonly known as "magueys") is a large group of succulent plant endemics of America that thrive in arid and semiarid environments [6]. *Agave* spp. are perennial plants that belong to the *Asparagaceae* family, with 210 species, where Mexico is considered the diversity center with 119 species [7]. The maguey contributes to the cultural essence of the country, and it plays an essential role in Aztec mythology [8]. So, agaves are traditionally cultivated in arid and semiarid areas throughout the country. The most extensively exploited species in Mexico are *Agave americana* L., *A. angustifolia* Haw., *A. cupreata* Trel. & A. Berger, *A. durangensis* Gentry, *A. funkiana* K. Koch & C. D. Bouché, *A. fourcroydes* Lem., *A. inaequidens* K. Koch, *A. karwinskii* Zucc., *A. lechuguilla* Torr., *A. marmorata* Roezl, *A. mapisaga* Trel., *A. maximiliana* Baker, *A. potatorum* Zucc., *A. rhodacantha* Trel., *A. salmiana* Otto ex Salm-Dyck, *A. sisalana* Perrine, and *A. tequilana* F. A. C. Weber [9]. However, it is estimated that many other species have not been classified up to now [10]. Many of the species grow wild; however, some of them are cultivated, such as *Agave angustifolia* Haw (espadín) in the state of Oaxaca, *A. salmiana* in San Luis Potosi and Zacatecas, *A. potatorum* in Guerrero, *A. cupreata* in Michoacán, and *A. duranguensis* (cenizo) in Durango [11].

The agaves are propagated and maintained by local producers who preserve the traditional knowledge associated with their different uses and purposes [12,13]. In this regard, the traditional selection of agave varieties could be divided into two periods. The first includes pre-Hispanic times, where the obtention of the optimal germplasm was ruled by selection to produce fibers and foods [including fermented beverages like pulque]. Secondly, modern selection started with distillation technology during the Spanish colonial times, and germplasm selection was ruled by the high sugar content required to produce distilled beverages [14].

The molecular evidence of the evolution of *Agave* spp. has been explored through phylogenomics studies and the traces of some evolutionary pathways based on comparative gene sequencing data [15]. Therefore, authors could carry out a phylogenetic reconstruction of the *Agave* genus by increasing the taxonomic sampling and selecting the appropriate molecular markers of 83 different *Agave* spp. These wild populations are genetically more diverse than the anthropogenic population with greater heterozygosity, polymorphic loci, effective number of alleles, and allelic richness. On the contrary, cultivated varieties show fixed heterozygosity for several loci in all populations [non-existent in wild populations], fewer multilocus genotypes, which differed by fewer alleles, and more significant differentiation among populations than was characteristic of wild populations [16].

**Figure 1:**
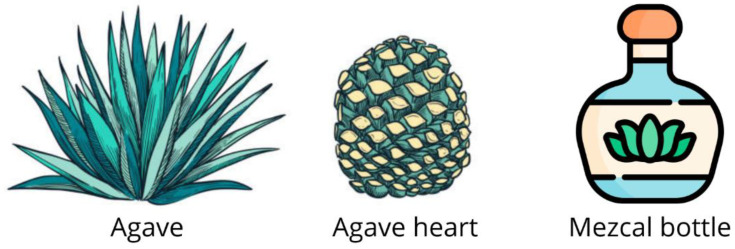
Agave and agave heart (after “jimado” or leaf removal).

The plants (see Figure 1) are perennial evergreen xerophytes, ranging from several centimeters to 4 m and producing large flowering stalks between 2 and 12 m tall after 5–15 years. The plants have a basal rosette of large stiff, lanceolate, succulent, and persistent leaves, often with a terminal spine and sometimes with spiny margins. In the transverse cross-section, the leaves are crescent-shaped. The epidermis is cuticularized, and the stomata are sunken. The stems are short and thick or basal, with leaves formed around the terminal meristem that they encircle. The shoots are typically monocarpic, i.e., they die after flowering. However, the side shoots may allow the plant to persist. The flowers are usually formed on a massive spike, sometimes termed a pole, and have a paniculate inflorescence [17].

*Agave* spp. has a specialized kind of photosynthesis known as crassulacean acid metabolism (CAM), thanks to which they can reach good productivity in arid areas [18]. Unlike C_3_ and C_4_ photosynthesis, the assimilation of CO_2_ during the CAM cycle occurs primarily at night when these plants open their stomata for gas exchange to take advantage of the lower temperatures and higher relative humidities, which help to reduce the rate of water loss due to evapotranspiration. CAM evolved from C_3_ photosynthesis in response to water and CO_2_ limitations [19]. CAM is a complex process that can be represented by several steps, as follows:

Night stage. Phosphoenolpyruvate (PEP) incorporates CO_2_ from the atmosphere using the enzyme PEP carboxylase (PEPC), leading to the formation of the four-carbon malic acid, which is stored in a vacuole.

Day stage. Malate is released and decarboxylated to produce pyruvate to be used in metabolism and CO_2_, which is fixed by the enzyme ribulose-1,5-bisphosphate carboxylase-oxygenase (RuBisCO) in the chloroplast during the Calvin cycle to generate two molecules of 3-phosphoglycerate (PGA). These molecules, in turn, are reduced to produce carbohydrates [20].

CAM plants can differ vastly in the degree to which they use CAM photosynthesis while keeping a fully functional C_3_ cycle in their photosynthetic cells, which affords flexibility in the amounts of dark and light CO_2_ uptake. The main CAM phenotypes include obligate (strong or constitutive) and facultative (inducible) species. Obligate phenotypes, with high nocturnal acid accumulation (ΔH^+^) and CO_2_ fixation, include the highly succulent agaves. Facultative phenotypes use the C_3_ mode of fixing CO_2_; they have no ΔH^+^ in the non-stressed state and little nocturnal CO_2_ fixation and ΔH^+^ during the C_3_ to CAM transition in the stressed state. These include a wide range of plant families, such as *Bromeliaceae*, *Cactaceae*, *Aizoaceae*, *Montiaceae*, *Lamiaceae*, *Vitaceae*, and *Didiereaceae* [21]. Increases in titratable acidity from dawn to dusk are in the range of 273 to 331 mmol H^+^ kg^−1^ of fresh mass in different species of *Agave* [22]. These increases in acidity (mainly malic acid) in the case of *Agave americana* L. have been related to high mitochondrial fluxes of carbon and electron transport that happen at night in CAM plants. High night levels of ascorbic acid, also present in *A. americana*, have been related to the necessity for antioxidant activity to eliminate the reactive oxygen species generated by high rates of respiratory electron transport that occur at night in CAM species [23].

Seeds do not propagate cultivated agaves because the plants are harvested before flowering. Moreover, the seeds usually are not viable for commercial production because they are sensitive to dry periods. Moreover, the seeds can also be problematic because of possible genetic variation. So, then, the most used method is vegetative propagation, where bulbils or suckers are used. Bulbils are young plantlets that occur on the inflorescence of mature plants. Propagation by removing the rhizome shoots from the mother plants is also common. Moreover, in vitro propagation of agave in a semi-solid culture medium has been widely studied in different agave species [24,25]. Before establishing an Agave plantation, the land should be cleared to remove the existing vegetation and stones, although mulching the vegetation by covering the land could also be used. Then, plants are transplanted from a nursery into 30 cm deep furrows [26]. The density varies between 5000 to 10,000 individuals per ha. Harvesting is performed when the plants are mature, which is specific for each individual, even in the same species and microclimate, at an age between 7 and 15 years [17,27,28].

The uses of *Agave* spp. include the production of fiber [leaves], food [baked leaves, stems and stalks, and boiled and cooked flower buds], beverages, such as pulque, mezcal, tequila, etc., medicine [leaves], construction [stalks], and live fences [29]. These agave products commonly offer goods for self-consumption, especially for people in arid and semiarid areas [30]. The preparation of fermented beverages [pulque] and distilled musts [mezcal, tequila, raicilla, bacanora] is derived from the fresh sap extraction, traditionally called aguamiel. The sap is collected with concave instruments that scrap the apical meristem where the sap accumulates [31,32].

In 2020, Mexico produced 1,913,026 tons of Agave from 25,741 Ha to 129,982 Ha of a total area dedicated to this succulent; of this, close to 80% was destinated to blue Agave for tequila production, and the rest was used for mezcal, pulque, and other products [33]. The national mezcal production in 2022 was 14,165,505 L, and it has been significantly increasing in the last decade. Oaxaca producers export around 8.5 million liters of mezcal to 81 different countries [34]. The Mexican government estimated that 5728 people were formally employed in the tequila and mezcal industries in 2019 [35]; however, most producers are informal employees. So, only in Oaxaca, 48,000 families are dedicated to the artisanal production of this spirit [34]. Then, most of the mezcal production is traditionally organized as a family activity, and the production and elaboration technologies have spanned generations for centuries. However, the growing national and global demand for this beverage is changing the production market, land use, and social relations in the producers’ communities [36].

## 3. Elaboration

Agave harvest. Mezcal is produced from at least 25 species of Agave. They are harvested after around eight years of development, their leaves are cut (“jimado”), and the stem or heart is transported for cooking. The composition (%) of the raw agave heart is as follows: moisture 65, crude fiber 11.91, fructans 18.11, reducing sugars 1.12, protein 0.13, and ash 2.76 [37].

### 3.1. Cooking

This process is performed in rustic ovens, where heat is provided by steam injection, or in pit ovens, where a fire is lit at the bottom of the pit before a layer of small stones is added. In this cooking step, the fructans contained in the agave are hydrolyzed into simple fermentable sugars, primarily fructose. Fructans are the most important reserve polysaccharides in agave plants, and their concentration is higher in the stem and base of the leaves. There are several studies about the extraction and characterization of the Agave plant fructans [38,39,40]. During the cooking step, two main reactions frequently occur as follows: hydrolysis of the fructans to oligosaccharides and monosaccharides and degradation of the monosaccharides to furfural and 5-[hydroxymethyl]furfural (HMF). The first reaction generates the fermentation substrates for the yeasts, but the second one generates products that have an inhibitory effect on yeast growth and ethanol production in fermentative processes. A kinetic study of the autoclave thermal hydrolytic process of *Agave salmiana* for mezcal production was developed by García-Soto et al. [41]. Their study included the reactions of hydrolysis and degradation. The results indicated that the thermal hydrolysis of agave was optimal, from the point of view of ethanol yield in the mezcal fermentation, in the temperature and in cooking time ranges of 106–116 °C and 6–14 h, respectively. These optimal conditions resulted in a fructan hydrolytic reaction of 80% and a furan concentration in the final syrup of only 1 g/L.

### 3.2. Milling

The juice of the cooked agave is usually extracted using rudimentary mills [named ‘‘tahonas’’], which have a 1.5 m diameter circular stone that rotates in a circular pit on the cooked agave [42]. Sometimes these mills are driven by mules or donkeys. Water is added during this milling process, which is very complex, and only a few studies are available [43]. One study concluded that the thickness of the stone is to be as big as possible, whereas the contact area must be as small as possible, to extract the maximum amount of juice. Each milling device has a different performance: while wooden mallets can grind between 100 and 150 kg of agave/h, the blade and Chilean mills can mill up to 1000 kg of agave/h [3]. All grinding processes generate similar average yields: 50.7 to 52.7% juice with a specific gravity of 1.108 and pH values from 4.2 to 4.6 [37]. These data can be translated into average juice extraction rates between 51.7 and 517 L/h, depending on the kind of mill used.

### 3.3. Fermentation

After the milling process, the resulting agave must, with or without dilution, be allowed to ferment, usually spontaneously for approximately 3–7 days. The physical, chemical, and microbiological average composition of the thermally hydrolyzed and unfermented agave juice is shown in Table 1. The fructose obtained by the hydrolysis of fructans is the main fermentable sugar, although glucose and some pentoses are also present in low concentrations.

Among a wide variety of compounds, which includes simple and complex carbohydrates, ethanol, methanol, higher alcohols, furfurals, terpenes, and Maillard products, several different organic acids produced by microbial metabolism have also been detected during the fermentation of the agave must (see Table 2). The presence of these compounds has been related to the final taste and aroma of mezcal [46,47]. The type and amount of these compounds depend on the composition of the must and the microbial communities that carry on the spontaneous fermentation of the mezcal.

There are many studies on the composition of these bacterial and yeast communities, and the results show enormous variations depending on several factors. Some of the criteria that the microbial strains must meet prior to their use in mixed cultures are fermentation capacity, ethanol tolerance, and cross-inhibition or killing activity. Table 3 shows the predominant bacteria and yeasts isolated from the fermentation process of mezcal according to different studies. Of the four studies that included the identification of bacteria, three of them isolated strains of *Lactiplantibacillus plantarum*, and two of them isolated strains of *Zymomonas mobilis*. *L. plantarum* is a lactic acid bacterium commonly found in plant habitats and includes many probiotic strains. *Z. mobilis* is a natural ethanologen with many desirable properties, such as high alcohol tolerance, a wide pH range for ethyl alcohol production (pH 3.5–7.5), and GRAS status [49]. *Z. mobilis* has been commonly isolated from pulque (a traditional Mexican non-distilled alcoholic fermented beverage) fermentation, where it has a prominent role in the production of ethanol along with yeasts [37]. *Z. mobilis* has the Entner–Doudoroff (E-D) pathway, the enzyme pyruvate decarboxylase, and two alcohol dehydrogenase isoenzymes for the fermentative production of ethanol and carbon dioxide from glucose [50]. Therefore, it is expected that in relation to mezcal, this bacterium has a similar role (ethanol production). In the case of yeasts, they possess the Embden–Meyerhof–Parnas (EMP) pathway, leading to the production of pyruvate which, in turn, is converted into ethanol and carbon dioxide. While glucose is the most common substrate, the adaptableness of yeasts allows them to be able to use various other carbohydrate sources, such as fructose and sucrose as carbon sources. Yeast develops optimally in slightly acidic environments, characteristically within the pH range of 4 to 6 [51]. In regard to mezcal, 11 studies have included a complete identification up to the subspecies level. Eight of them included strains of *Kluyveromyces marxianus* and *Saccharomyces cerevisiae*, and three of them included *Torulaspora delbrueckii*. These studies highlight the importance of non-*Saccharomyces* yeasts in mezcal fermentation. Some of these yeasts are important as valuable alternatives with biotechnological potential in the production of aromatic compounds (other than ethanol). A *Kluyveromices marxianus* strain isolated from the natural fermentation of *Agave duranguensis* for mezcal elaboration has been shown to produce aromatic compounds such as ethyl acetate, isoamyl acetate, 2-phenylethanol (2-PE) and 2-phenylethylacetate from glucose and fructans. The presence of these compounds is considered to make mezcal even more attractive [52]. In connection with *Saccharomyces cerevisiae*, the biosynthesis of 2-PE is associated with the Krebs cycle and the Shikimate and Ehrlich pathways. Although 2-PE can be de novo synthesized from glucose, the efficiency is quite low due to feedback regulations in many branched metabolic pathways, so its use for the production of aromatic compounds is not usually recommended unless genetically engineered strains are used [53]. A recent study [54] concluded that the main variable constantly associated with the composition of the bacterial and yeast populations was the distillery, suggesting that local elaboration practices and the characteristics of the geographical site have a strong influence on the microbiomes.

The natural fermentation of mezcal is a slow process, which can fluctuate from 8 to 30 days. This fermentation process is characterized by low ethanol yields, a fermentation efficiency of around 30 to 40%, and changing concentrations of residual sugars (10–60 g/L) [62]. The ethanol concentrations in the must have been reported to vary from 4 to 12.45% *v*/*v* [59]. Fermentation tanks vary in their shape, material [wood vessels, masonry vats, or stainless steel tanks], and capacity (500–10,000 L) [65]. There are some studies aimed at the optimization of the fermentation conditions in order to obtain the highest ethanol production, maximum product yield and productivity, and the best quality mezcal [66]. Ojeda-Linares et al. [2] performed a network analysis and found that in the peer-reviewed literature, mezcal is the most studied traditional Mexican beverage. They identified nine conceptual clusters in this subject, and they observed that in the mezcal cluster, *Agave potatorum* is the most studied species and *Saccharomyces cerevisiae* is the most characterized microorganism. In the case of the yeast cluster, they highlighted the use of non-*Saccharomyces* yeasts to improve the aroma profile and the ethanol production of mezcal.

### 3.4. Distillation

The distillation equipment varies depending on the mezcal category, as described before, and the process always includes a double distillation. All mezcals must have an ethanol content between 35 and 55% *v*/*v* at 20 °C. For centuries, and until recently, it was believed that the distillation process had been brought to the new world from Spain. However, recent investigations revealed archaeomagnetic evidence of the pre-Hispanic origin of mezcal, indicating that alcohol distillation was known in Mesoamerica long before the arrival of Europeans, for at least 2500 years. This evidence was found at the ceremonial and administrative center of Xochitécatl–Cacaxtla in the Mexican state of Tlaxcala, where several ovens with cooked maguey remains were revealed [67]. The efficiencies are usually between 10 and 20 kg agave for 1 L of mezcal, but, sometimes, higher values of up to 25–35 kg of agave can be obtained [3]. Currently, most mezcal is produced by means of traditional copper alembics, with a capacity from 300 to 800 L. The first distillation is carried out commonly by adding both the agave juice and bagasse into the still, obtaining an alcohol content by volume (ABV) in the must between 20 and 30%. This product is called “ordinario”. The second distillation is performed to achieve a 2–3-fold increase in ethanol concentration (between 50 and 60% ABV) in the “ordinario”. The distillate is fractionated into three cuts, known as heads, heart, and tails. The still pots are commonly heated with firewood where the amount and type of wood usually defines the rate of increase in temperature. However, the use of this method does not allow for a sensible control of heat, causing disparities in the separation of the compounds due to variations in temperature [68,69].

To obtain a product that achieves the specifications of the NOM standard, the heads and tails are removed during the second distillation. The behavior of volatile compounds during the distillation of mezcal was studied in detail by Nolasco-Cancino et al. [69]. They prepared mezcal from *Agave angustifolia* Haw. The first distillation was performed in a 300 L copper still containing 150 L of fermented must and 100 kg of bagasse at an average flow rate of 210 mL/min. In the second distillation, the flow rate was maintained at approximately 170 mL/min, and the distillate was separated into three fractions, i.e., heads, heart, and tails, which consisted of approximately 3.6% (9 L), 34.4% (86 L), and 4.8% (12 L), respectively, of the base “ordinario” volume (250 L) poured in the still. The alcohol concentration in the heads fraction ranged from 77.71 to 74.3% *v*/*v*; in the heart fraction, it ranged from 74.72 to 30.39% *v*/*v*, and in the tails fraction, it ranged from 30 to 14.4% *v*/*v*. The first liter of each cut fraction contained 1731.86, 656.54, and 102.6 mg/100 mL of a.a. (anhydrous alcohol) of esters; 421.21, 452.28, and 40.26 mg/100 mL a.a. of higher alcohols; 72.86, 35.37, and 1.77 mg/100 mL a.a. of aldehydes; 135.33, 142.95, and 247.6 mg/100 mL a.a. of methanol; and 0.30, 0.45, and 3.04 mg/100 mL a.a. of furfural. Higher alcohols, esters, and aldehydes predominate in the head fraction. The presence of higher alcohols, such as n-butanol, 2-butanol, isobutyl alcohol, amyl alcohol, and isoamyl alcohol, with high boiling points (>108 °C) in the heads fraction is probably due to their low water solubility and high affinity to ethyl-alcohol-forming azeotropes, which distill together with it. Higher alcohols form the largest group of aroma compounds in most spirits. Furfural and methanol prevail in the tails fraction. This behavior of methanol is possibly due to its high solubility in water and its capacity to form hydrogen bonds with this molecule to form clathrates of high molecular weight and decreased volatility [70]. With respect to esters, the Mexican standard NMX-V-005-NORMEX-2013 indicates that esters should be considered as the sum of ethyl acetate and ethyl lactate. In the second distillation, the heads fraction has a high concentration of esters (1731.86 to 883.77 mg/100 mL a.a.), mainly ethyl acetate. Esters are present in the heart fraction, ranging from 656.64 to 95.48 mg/100 mL a.a. (ethyl acetate varies from 471.92 to 26.38 mg/100 mL a.a. and ethyl lactate from 9.61 to 47.79 mg/100 mL a.a.). As the distillation process progresses, the ethyl acetate concentration continues to decrease while the ethyl lactate increases. Thus, the tails fraction consists mainly of ethyl lactate. The origin of the different volatile compounds is different. Aldehydes, such as butanal and 2-methyl butanal, are thermally generated during the first distillation, whereas acetaldehyde is synthesized by yeasts. Higher alcohols are also generated by yeasts from amino acids. Methanol is produced during agave heart cooking by the demethoxylation of the pectins in agave. Finally, the thermal hydrolysis of fructans during the cooking step also leads to the production of furans, such as 5-(hydroxymethyl) furfural, furfural, and others [69].

Recently, sustainable solar-powered approaches to distilling have started to be developed so that renewable sources of energy can be used in the production of spirits. As for mezcal, experimental solar stills, which use water heated at 90 °C by means of parabolic trough solar concentrators fed to copper alembics, have been used for the distillation of the product [71,72].

The average composition of mezcal, as shown in the Official Mexican Standard, can be seen in Table 4. The average composition of the fractions of the major and minor volatile compounds and esters are listed in Table 5 and Table 6.

These studies disclose the importance of knowing the behavior of the regulated chemical compounds indicated in the Standard during the distillation process to establish suitable cuts for the distillate. The relative abundance of the minor volatile compounds depends mainly on the strains of yeasts present in the must and the temperature of fermentation [44]. Additionally, there are studies in which the authors have searched for volatile compounds that could be used as identity markers due to the fact that they are present exclusively in only one distilled beverage and not in others. In the case of Mexico, these identity markers are 2-amino, n-butyl, 4,9-decadiene for tequila, 3-hidroxy, 2-butanone for sotol, and 5-methyl, 2-furancarboxaldehyde for mezcal [73].

### 3.5. Aging

Mezcal can be aged, depending on the class or style, in wood (oak) barrels for periods of up to one year; see the definitions in the previous subsection. The assessment of mezcal aging has been performed by a combination of Raman spectroscopy and multivariate analysis techniques. Raman spectroscopy is an analytical technique used to study the vibrational characteristics of molecular samples, which can be turned into data about molecular conformation. Multivariate analysis is achieved using principal component analysis discriminant analysis (PCA-DA) and partial least squares discriminant analysis (PLS-DA). The first principal component discriminates between aged and rested mezcal, while the second discriminates between white and matured (rested and aged) mezcal. Raman and multivariate analysis can distinguish between mezcal samples with different aging times (white, rested, and aged mezcal) and may offer an objective replacement for current sensory tests [74]. Distilled alcoholic beverages acquire an amber or yellowish color after variable periods of aging in wood barrels as a consequence of the extraction of phenolic compounds from the barrel wood. These compounds contribute to flavor and aroma. The phenol profile and antioxidant capacity of mezcal aged in oak wood barrels have been performed by HPLC/DAD and DPPH techniques, respectively. The results showed that the phenol profiles and contents were so specific for every stage of aging that they could be considered as valuable quality markers for the different classes of mezcal [75]. The sensory profile of mezcal is distinctive to each region due to the volatile compounds it contains [76], which are derived from the fermentation, such as ethyl acetate, ethanol, methanol, 3-methyl-1-butanol, propanol, 2-methyl-1-propanol, and acetic acid [47]. There are various techniques for volatile compound identification, which include simple colorimetric chemical methods, the already considered classical methods, such as Gas Chromatography (GC), solid-phase extraction or liquid–liquid extraction, and subsequent identification by gas chromatography coupled to mass spectrometry (GC-MS) [46,77,78]. In addition, the latest generation methods can increase the speed and accuracy of the analysis, such as direct injection electrospray ionization (DIESI) and low-temperature plasma ionization (LTP) [79]. The latter, based on the characterization and identification of the chemical fingerprint through mass spectrometry, allows rapid discrimination of the authenticity of distillates, such as tequila or mezcal. The working group of Vera-Guzmán [78] identified the compound 3-ethylphenol only in samples of *Agave angustifolía* mezcal. Therefore, they proposed that the presence of such a compound could be used as a marker of the authenticity of the mezcal prepared from this species. However, it would be necessary to carry out a study with more samples, such as those of *Agave angustifolia* mezcal from different regions [e.g., North, Central, and South]. As different working groups have reported, the differences in the volatile content are determined not only by the species but also by the process conditions, microbial strains, and distillation techniques used to obtain the distiller cuts (heads, heart, and tails). Distinctive sensory notes in mezcal usually include soil, smoke, and acidity [77].

**Figure 2:**
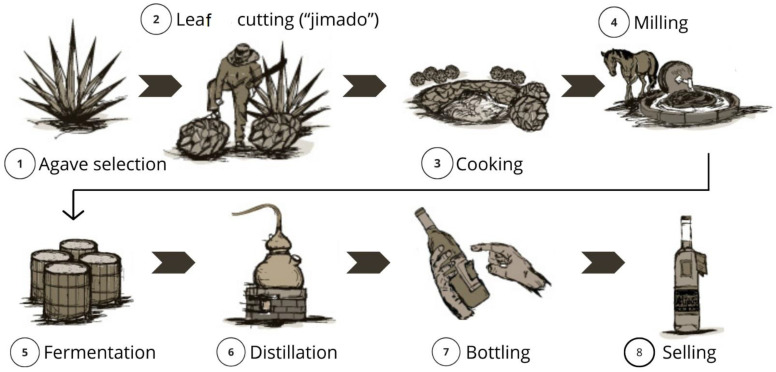
Flowchart showing the stages of elaboration of white mezcal; adapted from [80].

A process flowchart is shown in Figure 2.

## 4. Potential Health Benefits Related to Mezcal

### 4.1. Prebiotics

There are various reports about the prebiotic activity of agave fructans in several Agave species, such as *A. tequilana*, *A. salmiana*, *A. atrovirens*, and *A. angustifolia* Haw [38,40,81]. The fructans present in agaves, such as *A. tequilana* and *A. angustifolia*, were classified as graminans and agavins, and not as inulin [6,82]. These fructans (35–73%_d_._b_. of the Agave) [82], have a complex mixed branched structure with the presence of β(2 → 1) and β(2 → 6) bonds, which stimulate the growth of *Bifidobacterium* and *Lactobacillus* species and can help decrease the blood levels of glucose and cholesterol [83]. Therefore, these fructans have been considered as prebiotics since they stimulate the growth of beneficial bacteria in the colon, which, in turn, generates an improvement in the gastrointestinal health of the human host. The availability of this kind of fructan, to be used as prebiotics, depends strongly on the amount of wild agave plants since the cultivated ones are reserved for the preparation of tequila and mezcal. However, the mezcal industry generates byproducts, including leaves and bagasse, that can be re-valorized as a source of prebiotics. Bagasse is the fibrous residue obtained after the stem is used for juice extraction for mezcal elaboration and represents approximately 40% of the original stem weight. This byproduct is an excellent source of bioactive compounds (fructans, agavins, and phenolic compounds) that can be recovered and used in functional foods. Additionally, agavins increase short-chain fatty acids in the feces of obese mice, as well as decrease blood glucose and triglycerides [4,84]. The agave residual leaves from the mezcal industry also have an important fructan content (37%) [32,83]. On the other hand, Arrizon et al. [57] isolated sixteen yeasts belonging to *K. marxianus*, *T. delbrueckii*, and *C. apicola* species positive for fructosyl transferase activity. This enzyme (FTase; E.C. 2.4.1.9) catalyzes the formation of fructo-oligosaccharides (FOSs) from sucrose, so these yeast strains isolated from mezcal fermentation could be used for the in vitro synthesis of these FOSs, which are widely used in the food industry because of their techno-functional and prebiotic properties [85]. In the case of mezcal, fructans or FOSs are not present in the beverage; however, the phenolic compounds that are present in rested and aged products are currently being considered as a special class of prebiotics, which are discussed in Section 4.5.

### 4.2. Probiotics

The isolation, identification, and study of the probiotic potential of microorganisms from traditional fermented foods is one of the most important trends in food biotechnology research. Hernández-Delgado et al. [64] isolated potentially probiotic LAB from the fermented must of *Agave angustifolia* Haw used for the elaboration of mezcal in the state of Oaxaca. The strains *Lacticaseibacillus rhamnosus* LM07 and *L. plantarum* LM17 and LM19 showed resistance to simulated gastrointestinal conditions, anti-inflammatory properties in TNF-α-stimulated HT-29 cells, and antioxidant and anti-inflammatory properties when tested in vivo in a mouse model with induced chronic colitis. However, non-LAB probiotics are also very important. Such is the case of *Zymomonas mobilis*, an ethanol-producing bacteria present in mezcal must. De Aguiar Silva et al. [86] found that this probiotic has a beneficial effect in lowering the levels of serum cholesterol and its lipoprotein fractions and in regulating intestinal transit. This indicates that the fermented musts of different Agave species could be an important source of potentially probiotic bacteria.

### 4.3. Synbiotics and Postbiotics

The International Scientific Association for Probiotics and Prebiotics (ISAPP) published a consensus statement on the definition and scope of synbiotics, indicating that they may be formulated using two approaches [87]:

(a) Complementary synbiotics, which is a mixture of probiotics and prebiotics in which each works independently to accomplish one or more health benefits.
(b) Synergistic synbiotics, which is a mixture of a selectively utilized substrate and a live microorganism chosen for its ability to deliver a health effect. Components comprising synergistic synbiotics collaborate to generate resulting health benefits.

Buitrago-Arias et al. [88] showed that acetylated agave fructans are a good substrate for the probiotic yeast *Saccharomyces boulardii*. This result is a good example of synergistic symbiotics. Additional examples include the mixture of Agave fructans (*Agave salmiana*) and the probiotic bacteria *Lacticaseibacillus casei* SACCO BGP93 and *Bifidobacterium animalis* subsp. *lactis* SACCO BLC1 [89]. The system *Agave-L. casei* is considered a complementary synbiotic, while the system *Agave-B. animalis* is a synergistic synbiotic. Another example of a synergistic synbiotic is the system formed by agave fructans and *Limosilactobacillus reuteri* DSM 17,938. In this case, fructans can be utilized by the co-administered live microorganism (*L. reuteri*) since it can produce GH32 endo-fructanases and exo-fructanases [90,91,92]. From the above experiments, it is possible to conclude that agave-derived fructans (graminans and agavins) with prebiotic and anti-inflammatory functions can be incorporated into foods along with probiotics isolated from *Agave* to formulate health-promoting synbiotics.

### 4.4. Solid-State Fermentation of Agave Byproducts to Produce Metabolites with Potential Health Benefits

In Mexico, the generation of mezcal agave bagasse (AB) has increased considerably in recent years, given the growing global demand for this beverage; around 206,000 tons of AB are produced annually only from mezcal production. Despite the great potential of AB to produce high-value metabolites, its use is limited. Some efforts, however, have been made to use AB in the production of health-related compounds.

Ibarra-Cantún et al. [93] showed the presence of phenolic compounds and flavonoids with good antioxidant activity in the extracts of the solid-state fermentation of agave mezcalero (*Agave angustifolia* Haw) bagasse with *Pleurotus ostreatus*, a common edible mushroom. This indicates that even the residues of mezcal fermentation can be used to produce secondary metabolites with potential health benefits and mushrooms with good nutritional value.

On the other hand, the leaves of *Agave angustifolia* Haw are one of the main agrowastes [biowaste materials] engendered by the mezcal industry and can become an important source of bioactive compounds, such as phenolic compounds, glycosylated flavonoid derivatives, phytosterols, and saponins, which could be used in the food and pharmaceutical industries [94,95]. However, the leaves can also be valorized through their use in solid-state fermentation for the production of valuable metabolites in the health industry. Agave leaves generated by the mezcal and tequila industry have been used, with good results, as a substrate by *P. ostreatus* in solid-state fermentation for the production of the enzyme laccase [96]. Laccases (EC 1.10.3.2) are multicopper blue oxidases that couple the four-electron reduction of oxygen with the oxidation of a broad range of organic substrates, including phenols and polyphenols. Laccases have been used in the enzymatic synthesis of complex medical compounds, such as anesthetics, antibiotics, and anti-inflammatory and anticancer drugs. Additionally, laccase has been reported to possess significant HIV-1 reverse transcriptase inhibitory activity and capacity to fight aceruloplasminemia (a medical condition lacking ceruloplasmin, a multicopper serum ferroxidase that regulates iron homeostasis) [97].

Postbiotics, which include non-living microorganisms or their constituents, have lately gained significant attention for their potential health benefits. Extensive research on postbiotics has revealed many beneficial effects on hosts, including antioxidant and antimicrobial activities, immunomodulatory effects, gut microbiota modulation, and enhancement in epithelial barrier function. Although these features resemble those of probiotics, the stability and safety of postbiotics make them an attractive alternative. Furthermore, by substituting postbiotics for antibiotics, it is possible to promote health and productivity while minimizing adverse effects. In regard to the mezcal process, many postbiotics remain in the residue of the first distillation and can be recovered and tested for different health-related bioactivities [98].

### 4.5. Alcohol and Polyphenols

The regular consumption of ethyl alcohol in moderation (one or two drinks per day) is broadly recognized to reduce overall mortality, mainly by lowering the risk of coronary heart disease [99]. These effects can essentially be attributed to the ability of alcohol to raise the plasma concentration of high-density lipoproteins and to decrease blood coagulability [100]. Therefore, the consumption of up to two mezcal drinks could be beneficial for health. Additionally, many aged distilled alcoholic beverages have a high total antioxidant capacity. This effect can largely be ascribed to the presence of phenolic compounds [101]. Ellagic acid is the phenolic present in the highest concentration in most aged spirits. Moderate amounts of syringaldehyde, syringic acid, and gallic acid, as well as lesser amounts of vanillin and vanillic acid, are quantifiable in most samples of whiskey, brandy, and rum but are largely undetectable in the case of white rum, gin, and vodka [100]. This phenol-related effect has been tested in tequila, where a good antioxidant capacity was detected (3.1 mg GAE/L), which was attributed mainly to the presence of caffeic acid (18.6 mg/L) followed by 2-furaldehyde (1.9 mg/L), ellagic acid (0.9 mg/L), syringaldehyde (0.5 mg/L), syringic acid (0.2 mg/L), and gallic acid (0.03 mg/L) [102]. Regarding mezcal, the main phenolic compound found in young (46 days of aging) and reposado (206 days of aging) mezcal was a hydroxycinnamic acid derivative at concentrations of 34.39 and 43.89 mg/L, respectively. The changes in phenolic compounds during aging can be seen in Table 7. It can be observed that the longer the time, the larger the number of detected compounds. The concentration of total phenols varies from 214.24 (47 days) to 852.38 mg/L (206 days). Mezcal samples are effective antioxidants as free radical scavengers, with higher levels of antiradical activity at any time, between 47 and 206 days [75].

All these polyphenols and color compounds are readily extractable by spirits during wood cask maturation [103] or during aging in different kinds of wooden fragments [oak, acacia, chestnut, etc.] [104]. A review of the main polyphenols present in oak woods was published by Zhang et al. [105], indicating that these important phytochemicals show potential protective factors against cancer and heart diseases and also a good antimicrobial effect. Polak et al. [106] determined the Trolox equivalent antioxidant capacity (TEAC) of different alcoholic beverages using an electron paramagnetic resonance technique. They concluded that the TEAC value significantly depends on the total phenolic content, production method, and raw material used to produce the alcohol. In their study, it was presumed that the TEAC value of alcoholic beverages is independent of the alcohol content, and they reported values of 0 for white spirits (vodka or in this case white mezcal) to 13.6 μM/100 mL for one-year-aged spirits (common brandy or, in this case, “añejo” mezcal). Referring to whiskey, Koga et al. [107] reported that the free-radical scavenging molar activity of the main phenolic compounds [ellagic and gallic acids and lyonil resinol] was 1.68 to 3.14 times that of Trolox. It is worth mentioning that polyphenols are present only in “reposado” and “añejo” mezcals; however, some infused mezcals can contain some phytochemicals with antioxidant and antimicrobial properties. This is the case, for example, of the mezcal abocado con cedrón [mezcal infused with lemon verbena or *Aloysia citrodora*]. This aromatic herb contains the acyclic monoterpene aldehyde in its essential oil, which is known as citral. This essential oil is a GRAS food additive with antioxidant and antimicrobial properties. In the Mexican market, there are many kinds of infused mezcals (abocado) with different regional traditional medicinal herbs [108].

Recently, a new concept of second-generation synbiotics has been developed, which claims that prebiotics could be best defined based on their physiological effects or functional properties in the host rather than their specificity for a determined probiotic microorganism. Plant polyphenols are quickly evolving as good candidates for this definition. These second-generation prebiotics would offer strong health benefits in addition to nonspecific modulation of the host microbiome. As for vegetable polyphenols, the microbial biotransformation-mediated production of new bioactive molecules, and the resulting intensification in bioavailability, may result in synergistic (i.e., second-generation) effects of the polyphenol–probiotic combination, which would have a higher bioactivity and cellular effects compared with that of its individual components. Many of the polyphenols present in rested, aged, and abocado mezcals are strong candidates to be called second-generation synbiotics [109]. Additionally, there are other approaches that can be used to understand the capacity of polyphenols to promote the proliferation of beneficial gut bacteria through their direct and collaborative bacterial utilization and their inhibitory action on potential pathogenic microorganisms. The term *duplibiotic* has been proposed to describe an unabsorbed substrate (such as polyphenols) that modulates the gut microbiota by both antimicrobial and prebiotic modes of action. The duplibiotic effect of polyphenols could participate in reducing metabolic trouble and gut dysbiosis, positioning these compounds as dietary strategies with therapeutic potential [110]. Again, this new concept can easily include the polyphenols in mature mezcal.

## 5. Final Remarks

In summary, this review emphasizes the current state of the art in the emerging field of mezcal and includes the official definitions used in the industry, characteristics of *Agave* species and varieties, mezcal elaboration technology, and potential health benefits related to mezcal, including prebiotics, probiotics, synbiotics, and bioactive compounds, such as phenolics and ethyl alcohol. Although research on mezcal is not recent, the application of the principles of biotechnology is relatively new and still faces several challenges. The microbiome of each distillery is unique and is correlated with the properties of the corresponding final product. However, from all this research, it can be concluded that mezcal is a beverage that may have many potential health benefits when drank in moderation, and some of its byproducts, such as the leaves and fermentation residues (bagasse), can be used as a source of bioactive compounds and probiotic microorganisms. Additionally, the mezcal industry uses fermentation and distillation processes with a great amount of technological improvement opportunities in the processes themselves and in the quality control areas. More research on this important beverage is necessary since the trend in the field of mezcal research is still growing, mainly in the biology, biotechnology, and health benefits aspects.

## Figures and Tables

**Table 1:** Physical, chemical, and microbiological average composition of cooked unfermented agave juice.

Component or Parameter	Concentration or Value	Reference
Specific gravity	1.107–1.108	[37]
Glucose	1.3–3.9%	[37]
Fructose	13.7–17.8%	[37]
Direct reducing sugars	16.7–18.9%	[44]
Total carbohydrates	24.2–24.8%	[44]
Assimilable nitrogen	0.02–0.03%	[37,45]
pH	4.2–4.6	[41,45]
Total yeast	<1 × 10^6^ cfu/mL	[45]

**Table 2:** Composition of spontaneously fermented *A. angustifolia* Haw juice for mezcal production [48].

Compound	Concentration
Direct reducing sugars (%)	28.2–79.0
Protein (%)	0.072–0.11
Ethanol (% *v*/*v*)	4.2–5.0
Ethyl acetate (mg/100 mL a.a.)	1.4–3.45
Methanol (mg/100 mL a.a.)	8.87–10.71
1-Propanol (mg/100 mL a.a.)	0.59–0.88
2-Methyl propanol (mg/100 mL a.a.)	2.59–2.84
3-Methyl butanol (mg/100 mL a.a.)	8.18–8.64
*n*-Butanol (mg/100 mL a.a.)	0.004–0.014
Acetic acid (mg/100 mL a.a.)	40.46–65.05

a.a.—anhydrous alcohol.

**Table 3:** Predominant bacteria and yeasts isolated from the fermentation process of mezcal.

Bacteria	Yeasts	Agave Species and State in Mexico	Reference
*Zymomonas mobilis* subsp. *Mobilis*, *Z. mobilis* subsp. *Pomaceae*, *Weissella cibaria*, *W. paramesenteroides*, *Lactobacillus pontis*, *L. kefiri*, *L. plantarum*, *L. farraginis*	*Clavispora lusitaniae*, *Pichia fermentans*, *Kluyveromyces marxianus*	*A. salmiana* from San Luis Potosí	[55]
*Pediococcus parvulus*, *L.* *brevis*, *L. composti*, *L. parabuchneri*, *L. plantarum*, *Weissella* sp., *Bacillus* sp.	NR	*Z. angustifolia*, *A. lechugilla* [Torr] and *A. americana* *(montium sancticaroli)* from Tamaulipas	[47]
NR	*K. marxianus*	*Agave* sp. from Guerrero	[56]
NR	*Saccharomyces cerevisiae*, *K. marxianus*, *P. kluyveri*, *Zygosaccharomyces bailii*, *Cl. Lusitaniae*, *Torulaspora delbrueckii*, *Candida ethanolica*, *S. exiguus*	*A. salmiana* from San Luis Potosí	[42]
NR	*Z. bisporus*, *T. delbrueckii*, *K. marxianus*, *C. apicola*, *C. zemplinina*, *C. boidinii*, *P. anomala*, *Schizosaccharomyces pombe*, *Rhodotorula mucilaginosa*, *Hanseniaspora osmophila*, *Dekkera anómala*, *Issatchenkia orientalis*	*A. angustifolia* Haw, *A. potatorum*, and *A. karwinskii* from Oaxaca	[57]
NR	*S. cerevisiae*	*A. cupreata* from Michoacán	[58]
NR	*S. cerevisiae*, *K. marxianus*, *T. delbrueckii*, *C. diversa*, *P. fermentans*, *H. uvarum*	*A. duranguensis* from Durango	[11]
NR	*S. cerevisiae*, *K. marxianus*, *K. marxianus*, *var. drosophilarum*	*A. cupreata* from Michocán	[59]
*Z. mobilis*, *L. casei*, *L. harbinensis*, *Acetobacter senegalensis*, *Komagataeibacter saccharivorans*, *Bacillus subtilis*, *B. pumilus*, *Kocuria rhizophila*	*S. cerevisiae*, *Z. rouxii*, *Z. bisporus*, *T. delbrueckii*, *P. membranifaciens*	*Agave* sp. from Oaxaca	[60]
NR	*S. cerevisiae*, *K. marxianus*, *Z. bailli*, *Z. rouxi*, *P. kluyveri*, *I. terrícola*	*A. cupreata* from Michoacán	[61]
NR	*P. kudriavzevii*, *P. manshurica*, *S. cerevisiae*, *K. marxianus*	*A. angustifolia* Haw from Oaxaca	[62]
NR	*S. cerevisiae*	*A. angustifolia* Haw from Oaxaca	[63]
*L. plantarum*, *L. rhamnosus*, *Enterococcus faecium*, *Lactococcus lactis*	NR	*A. angustifolia* Haw from Oaxaca	[64]

NR: not reported.

**Table 4:** Mezcal composition according to the Official Mexican Standard NOM-070-SCFI-2016 [4].

Compound	Concentration Range
Ethanol (% *v*/*v*)	35–55
Dry extract (g/L)	0–10
Higher alcohols (mg/100 mL a.a.)	100–500
Methanol (mg/100 mL a.a.)	30–300
Furfural (mg/100 mL a.a.)	0–5
Aldehydes (mg/100 mL a.a.)	0–40

a.a.—anhydrous alcohol.

**Table 5:** Main volatile compounds in *A. angustifolia* Haw mezcal [69].

Compound	Concentration (mg/100 mL a.a.)
Ethyl acetate	89.17
Ethyl lactate	20.95
2-Butanol	0.79
*n*-Propanol	23.38
Isobutanol	57.15
*n*-Butanol	0.42
Isoamyl alcohol	221.68
Amyl alcohol	0.85
Methanol	150–250
Furfural	0.5–4
Acetaldehyde	8

a.a.—anhydrous alcohol.

**Table 6:** Minor volatile compounds and esters present in commercial mezcal [44].

Compound	Concentration (Relative Abundance Units)
2-methyl-1-propanol	3.90
3-methyl-1-butanol	23.88
Acetic acid	65.38
1-(2-furanyl)-ethanone	1.24
α-Terpineol	6.33
Phenyl ethyl alcohol	7.66
Phenyl ethyl acetate	4.94
3-methyl-1-butanol acetate	3.08
Caproic acid EE	4.07
2-hydroxy-propionic acid, EE	29.77
Caprylic acid, EE	18.62
Lauric acid, EE	4.45
Palmitic acid, EE	1.32

EE—ethyl ester.

**Table 7:** Phenolic compound concentrations (mg/L) in mezcal at different times of aging in oak wood barrels.

Phenolic Compound	47 Days	75 Days	110 Days	131 Days	175 Days	206 Days
Syringic acid	n.d.	n.d.	31.27	33.91	35.99	44.89
Phenolic acid	34.17	34.54	35.53	35.96	38.01	42.69
Hydroxycinnamic acid derivative	34.39	35.34	34.92	35.93	37.98	45.56
Flavona glycoside	n.d.	n.d.	n.d.	34.78	36.96	40.24
Benzoic acid	n.d.	n.d.	n.d.	n.d.	n.d.	38.54
Sinapic acid	n.d.	n.d.	n.d.	n.d.	n.d.	37.88
Luteolin glycoside	n.d.	n.d.	n.d.	n.d.	n.d.	38.27
Benzoic acid derivative	n.d.	n.d.	n.d.	37.06	38.65	43.89
Dihydroflavonoid	n.d.	n.d.	n.d.	n.d.	36.67	39.27
Hydroxycinnamic acid derivative	n.d.	n.d.	n.d.	n.d.	n.d.	40.61
Benzoic acid derivative	n.d.	n.d.	n.d.	n.d.	n.d.	37.59

n.d.—not detected.

## Data Availability

No new data were created or analyzed in this study.
